# Metabolomic analysis for asymptomatic hyperuricemia and gout based on a combination of dried blood spot sampling and mass spectrometry technology

**DOI:** 10.1186/s13018-023-04240-3

**Published:** 2023-10-11

**Authors:** Shanshan Liu, Yongting Liu, Xue Wu, Zhengqi Liu

**Affiliations:** 1 Guizhou University of Traditional Chinese Medicine, Guiyang, 550025 Guizhou China; 2 The Second Affiliated Hospital of Guizhou University of Traditional Chinese Medicine, Guiyang, 550003 Guizhou China

**Keywords:** Asymptomatic hyperuricemia, Gout, Mass spectrometry, Metabolomics, Biomarker, Dried blood spot

## Abstract

**Background:**

Gout is the most common inflammatory arthritis and closely related to metabolic syndrome, leading to excruciating pain and the decline in quality of patients’ life. However, the pathogenesis of gout is still unclear, and novel biomarkers are demanded for the early prediction and diagnosis of gout.

**Objective:**

This study aimed at profiling the dysregulated metabolic pathways in asymptomatic hyperuricemia (AHU) and gout and elucidating the associations between AHU, gout and metabolomics, which may aid in performing gout screening.

**Methods:**

A total of 300 participants, including 114 healthy controls, 92 patients with AHU, and 94 patients with gout, were analyzed by using a combination of dried blood spot (DBS) sampling and mass spectrometry (MS) technology. Multiple algorithms were applied to characterize altered metabolic profiles in AHU and gout. The mainly altered metabolites were identified by random forest analysis.

**Results:**

There were significant differences in AHU and gout compared with control group. The altered metabolites were involved in oxidation of fatty acids, carnitine synthesis, urea cycle, and amino acid metabolism in AHU and gout. Random forest classification of 16 metabolites yielded 3 important features to distinguish gout from AHU.

**Conclusions:**

Distinct metabolomic signatures were observed in AHU and gout. The selected metabolites may have the potential to improve the early detection of gout.

**Supplementary Information:**

The online version contains supplementary material available at 10.1186/s13018-023-04240-3.

## Introduction

Gout, the most common inflammatory arthritis, is induced by the deposition of monosodium urate (MSU) crystals in joints and soft tissues or the supersaturation of uric acid (UA) in extracellular fluid [1]. It has been reported to affect up to 6.8% worldwide human population [2]. In china, approximately 1–3% of people were affected, and the number is on the rise year by year [3]. Gout causes excruciating pain, and lead to the decline in quality of patients’ life. Hence, gout has been became to an important public health issue.

It is described as a typical progression from hyperuricemia without MSU crystal deposition, to MSU crystals without signs of gout, to acute gout flares, and to advanced gout [4]. Hyperuricemia is a common condition where high levels of serum uric acid (SUA) are present. It has been recognized as an important causal precursor during the development process of gout and can significantly increase risk of incident gout, and many individuals suffering from hyperuricemia form MSU crystals and develop gout, however, some of those individuals do not follow the trend, up to 76% of patients with asymptomatic hyperuricemia (AHU) could not detect MSU crystal deposition, and 10% of patients with AHU will not suffer from gout for life [5]. This discrepancy have led to many debates for the roles of SUA in the development of gout [6]. As such, it is a clinical challenge to find some other biomarkers for predicting who will develop gout.

Hyperuricemia and gout are closely related to metabolic syndrome, kidney injury, and cardiovascular disease [7–9]. Systematic metabolic changes were observed in both of the two diseases [10, 11]. However, it is still unsure what are the differences in metabolism of hyperuricemia and gout and whether these differences could affect acute gouty attacks.

Metabolomics is a rapidly developing filed and can investigate the quantity and type of molecules in organisms. The use of this technology can benefit to understand biochemical status in a system and monitor organism-wide alterations in metabolome [12, 13]. It can be widely used in disease diagnosis and detection, and is valuable to clarify pathogenesis of disease [14]. Such metabolomic methods have been used in the study of rheumatic diseases [15], however, limited studies were found in hyperuricemia and gout [16]. Recently, a metabolomic study revealed 23 different metabolites by gas chromatography (GC)/liquid chromatography (LC)-mass spectrometry (MS) method for 150 serum samples of acute gout arthritis (AGA), AHU and healthy controls [17]. Another study in larger cohorts has been conducted to profile metabolic alterations and disordered metabolic pathways for hyperuricemia and gout by LC–MS analysis, and further discovered 13 metabolites to distinguish hyperuricemia and gout from normouricemic controls [18]. However, most of these study were focus on male population. In order to extend results to female population, our study included female participants to further systematically profile metabolome for finding potential biomarkers to distinguish AHU from gout in a large cohort. Dried blood spot (DBS) sampling has been introduced as a microvolume sampling technique. It offers a simpler storage and easier transport compared with conventional whole blood sampling, and reduces infection risk by infectious pathogens. The combination of DBS sampling and MS technology can provide a high-throughput, reliable, and stable detection for a broad array of analytes, and has been used in newborn screening and selecting high sensitivity and specificity metabolic biomarkers for some kinds of cancer and cardiovascular diseases [19–21]. However, this technology were seldom used in the study of gout. As we all known, amino acids take part in the synthesis, excretion, and regulation of UA [22]. In addition, acylcarnitines showed associations with incident hyperuricemia [23]. In this study, we applied a combination of DBS sampling and MS technology to study AHU and gout. Altered levels of amino acids, carnitine/acylcarnitines and their ratios were determined for AHU, gout, and healthy controls. After systematic selection, 16 metabolites were significantly altered between AHU and gout groups. Among these metabolites, 3 metabolites were subsequently selected for distinguishing gout from AHU by random forest analysis.

## Materials and methods

### Study participants

In this study, a total of 300 participants were recruited, including 114 healthy individuals as control group, 92 patients with AHU, and 94 patients with gout, at the dedication of the Second Affiliated Hospital of Guizhou University of Traditional Chinese Medicine. Demographic data and clinical data were got from all participants (Table 1). The healthy participants met the following criteria: (1) age higher than 18 years; (2) levels of SUA were lower than 420 µmol/L in man and 360 µmol/L in woman; (3) without any clinically detectable severe diseases. All the patients with AHU had SUA levels higher than 420 µmol/L in man and 360 µmol/L in woman on 2 different days without the history of acute attack of gout and receiving drug treatment. All the patients with gout were in acute attack and diagnosed based on the classification criteria approved by the American College of Rheumatology Board and the European League Against Rheumatism Executive Committee in 2015 [24]. Participants with other diseases which could influence biological indicators were excluded, such as diabetes mellitus, hypertension, hyperthyroidism, hyperlipidemia, alcohol abuse, rheumatoid arthritis, or tumor. All participants were willing to participate in this study, and written informed consent was obtained. The present study was conducted under the Declaration of Helsinki, and followed the Ethics Committee of the Second Affiliated Hospital of Guizhou University of Traditional Chinese Medicine.

**Table 1:** Clinicopathologic characteristics of control, AHU, and gout groups

Characteristics	Control	AHU	Gout	*p*-value
Total number	114	92	94	
Gender				
Male	98	82	88	0.2048
Female	16	10	6	
Age (years)	47.325 ± 13.850	46.587 ± 14.425	50.830 ± 14.878	0.0875
BMI (kg/m^2^)	24.229 ± 4.034	25.207 ± 4.372	24.150 ± 4.023	0.1284
Blood pressure				
Systolic (mmHg)	115.044 ± 14.848	129.337 ± 17.580^†^	128.787 ± 14.659^†^	< 0.0001
Diastolic (mmHg)	74.132 ± 8.825	79.924 ± 11.562^†^	81.915 ± 11.486^†^	< 0.0001
Uric Acid (μmol/L)	288.921 ± 83.550	489.924 ± 69.982^†^	463.691 ± 122.274^†^	< 0.0001
eGFR (ml/min.1.73m^2^)	105.105 ± 8.691	99.348 ± 22.161	87.936 ± 27.375^†‡^	< 0.0001
AST/ALT	1.125 ± 0.208	1.025 ± 0.745^†^	1.013 ± 0.744^†^	< 0.0001
Total cholesterol (mmol/L)	4.281 ± 0.905	4.996 ± 1.317^†^	4.919 ± 1.275^†^	< 0.0001
Triglyceride (mmol/L)	1.024 ± 0.383	2.713 ± 3.646^†^	2.402 ± 1.238^†‡^	< 0.0001
HDL (mmol/L)	1.390 ± 0.164	1.202 ± 0.409^†^	1.124 ± 0.343^†^	< 0.0001
LDL (mmol/L)	3.002 ± 1.291	3.183 ± 1.105	3.214 ± 1.059	0.6210

AHU: asymptomatic hyperuricemia, BMI: Body-Mass Index, AST: aspartate aminotransferase, ALT: alanine aminotransferase, eGFR: estimated glomerular filtration rate, HDL: high density lipoprotein, LDL: low density lipoprotein

^†^*p* < 0.05 versus control group

^‡^*p* < 0.05 versus AHU group

### Blood sample collection and pretreatment

Labeled amino acid and carnitine/acylcarnitine internal standards with pure methanol were mixed together. Mixture of these dissolved isotope standards aimed at preparing stock solutions. Stock solutions were stored at 4 °C. Working solution was prepared by 100-fold dilution of stock solution. In quality control (QC) process, mixture of equal volumes from all collected samples was used as a pooled QC sample.

Blood samples were taken from all participants after an overnight fasting aimed at eliminating disturbance of diet. The blood samples for patients with gout were collected before medical treatments. A drop of whole blood was collected on DBS card, and then a disk with 3 mm diameter was punched from this card. The disk was placed into Millipore MultiScreen HV 96-well plate (Millipore, Billerica, MA, USA) in order to extract metabolites. A 100-µL working solution was added into a well containing a punched disk, subsequently, a 20-min gentle shaking was performed. After centrifuged at 1500 rpm for 2 min, the filtrate was transferred to a new flat-bottom 96-well plate. Aimed at monitoring the stability of MS detection, 2 low-level and 2 high-level QC sample solutions were randomly added into 4 blank wells. After dried in pure nitrogen gas flow at 50 °C, these filtrate and QC solutions were derivatized by using 60 µL mixture of acetyl chloride/1-butanol (10:90, v/v) at 65 °C within 20 min. After dried again, a mixture of each dried sample and a 100-µL mobile phase solution was prepared for the following metabolomics analysis.

### Metabolomics analysis

The direct injection MS was carried out on an AB Sciex 4000 QTrap system (AB Sciex, Framingham, MA) with an electrospray ionization source. MS analysis was performed under positive mode. The 20-µL sample was injected into this system. The mobile phase was 80% acetonitrile aqueous. The initial flow rate was 0.2 mL/min, decreased to 0.01 mL/min in 0.08 min, and then remained stable over the next 1.5 min. In the next step, flow rate was returned to 0.2 mL/min in 0.01 min and maintained for 0.5 min. The MS detection parameters were set as follows: ion spray voltage, 4.5 kV; curtain gas pressure, 20 psi; auxiliary gas temperature, 350 °C; sheath and auxiliary gas pressure, 35 psi. Scan modes and parameters were set according to a previous study [25]. Analyst 1.6.0 software (AB Sciex) was applied to control system, align spectrum, and gather amino acid and carnitine/acylcarnitine data. ChemoView 2.0.2 (AB Sciex) was carried out for data preprocessing.

### Data analysis

**Fig. 1:**
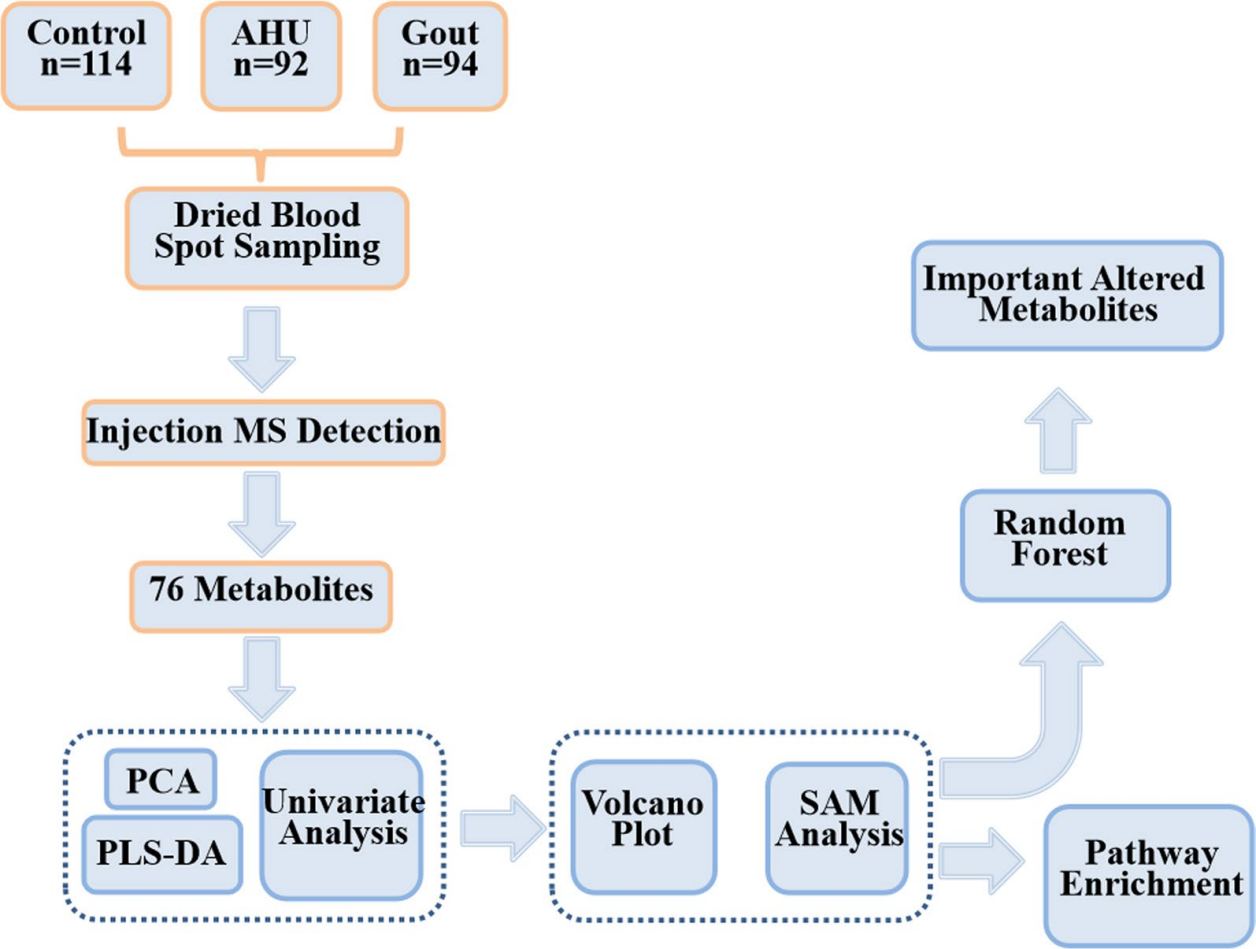
Design and data analysis for this study. AHU, asymptomatic hyperuricemia; MS, mass spectrometry; PCA, Principal component analysis; PLS-DA, Partial least squared discriminant analysis; SAM, Significance analysis of microarrays

The study design and workflow of data analysis are shown in Fig. 1. Principal component analysis (PCA), an unsupervised multivariate analysis, was used to discover general trends toward metabolomics data. In addition, a supervised partial least squares discriminant analysis (PLS-DA) model was built to distinguish AHU and gout from control group and obtain variable importance in projection (VIP) values which are the important variables contributing to the classification. In addition to that, the risk of over-fitting of PLS-DA model was evaluated by using a 200-times permutation test. For parametric variables, a t-test statistical analysis was used to assess whether the means of control, AHU, and gout groups were statistically different from each other, and ANOVA test was applied to compare the means of the three groups. Subsequently, Wilcoxon–Mann–Whitney test and Kruskal–Wallis test were performed for nonparametric variables. Benjamini–Hochberg procedure was executed to control false discovery rate (FDR) for hypothesis tests [26]. Volcano plots were created to find significantly altered metabolites with VIP > 1, fold change (FC) > 1.2 or < − 1.2, and adjusted *p*-value < 0.05 between two groups. Significance analysis of microarrays (SAM) was used to further select metabolites. MetaboAnalyst 5.0 online server was used to analyze metabolic pathway based on the significantly altered metabolites between two groups. Random forest approach is a classifier which contains a set of decision trees. It (R package rfPermute) was used to further extract metabolites based on the importance of all the metabolites. The response variable was permuted 5000 times in order to estimate *p* values for random forest importance. Statistical analysis was executed by using SAS software and R language.

## Results

### Demographics and clinical characteristics of enrolled participants

Demographics and clinical characteristics of all participants were collected, as shown in Table 1. A total of 300 participants, including 114 healthy individuals, 92 patients with AHU, and 94 patients with gout, were recruited in this study to define lbiomarkers candidates. There were 16 females in control group, 10 females in AHU group, and 6 females in gout group. No statistical differences was observed for gender, age, Body-Mass Index (BMI), and low density lipoprotein (LDL) among the three groups. Although patients with hypertension were excluded, systolic blood pressure (SBP) and diastolic blood pressure (DBP) in AHU and gout groups were significantly higher than control group. The levels of SUA in patients with AHU and patients with gout were distinctly higher than healthy individuals. In addition to that, patients with AHU have significantly higher levels of UA compared with patients with gout. The aspartate aminotransferase (AST)/alanine aminotransferase (ALT) ratio, total cholesterol, triglyceride, and high density lipoprotein (HDL) were significantly different in AHU and gout compared with control group. Except for estimated glomerular filtration rate (eGFR) and triglyceride, the other characteristics were not significantly different between AHU and gout.

### Metabolic profiles of control, AHU, and gout groups

**Fig. 2:**
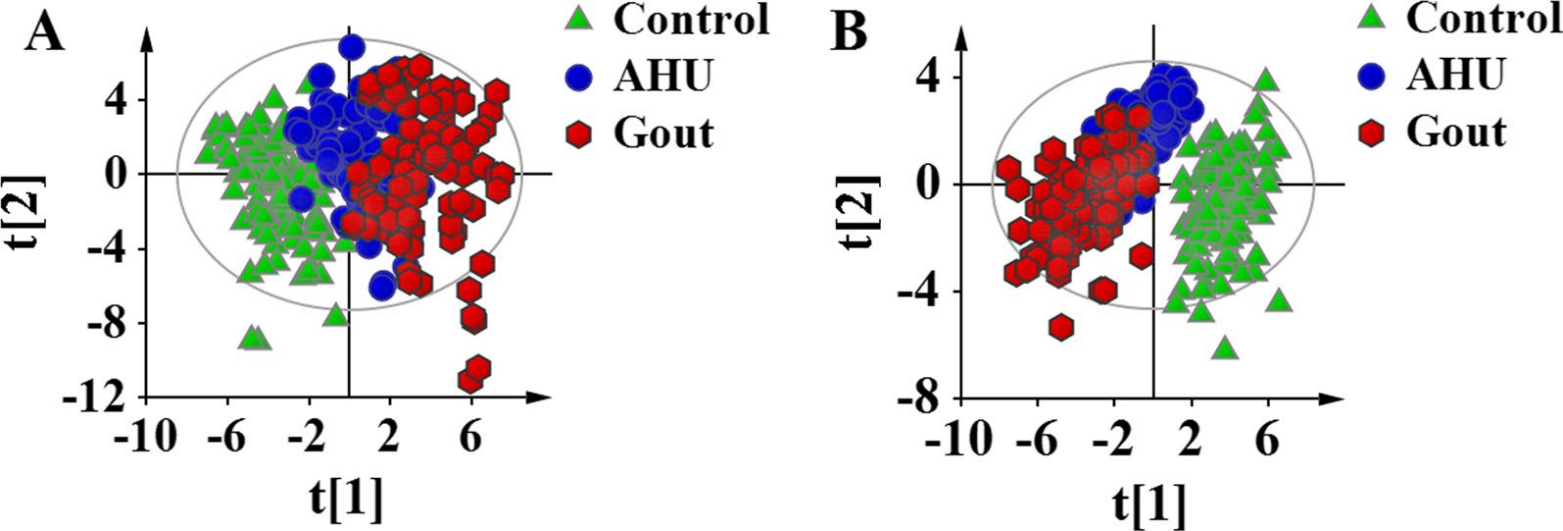
Score plots of PCA and PLS-DA analyses based on the 76 metabolites for AHU, gout, control groups. **A** Score plot of PCA analysis, indicating the separating trend among the three groups. **B** Score plot of PLS-DA analysis, suggesting the differential metabolic profiles among the three groups. The colors and shapes represent the participants from the three groups. AHU, asymptomatic hyperuricemia

We performed metabolomic analysis by using a combination of DBS sampling and direct injection MS technology to detect 76 metabolites, including 21 amino acids, 27 carnitine/acylcarnitine, and 28 related ratios. The detected metabolites in this study were described in Additional file 1: Table S1. An unsupervised PCA method was used to assess general trends toward these detected metabolites (Fig. 2A). The metabolites gave better separation between AHU or gout and control group, suggesting the metabolic alterations in AHU and gout compared with control group. In addition to that, similar separation trend was observed between AHU and gout. In order to further insight into metabolomic profiles, a supervised PLS-DA was carried out to maximize classification between the three groups (Fig. 2B). A 200-times permutation test ensured that this model was not over-fitting, as shown in Additional file 1: Figure S1. This analysis revealed that the detected metabolomic profile could distinguish control, AHU, and gout groups.

### Comparison of metabolic profiles between patients with AHU and control group

**Fig. 3:**
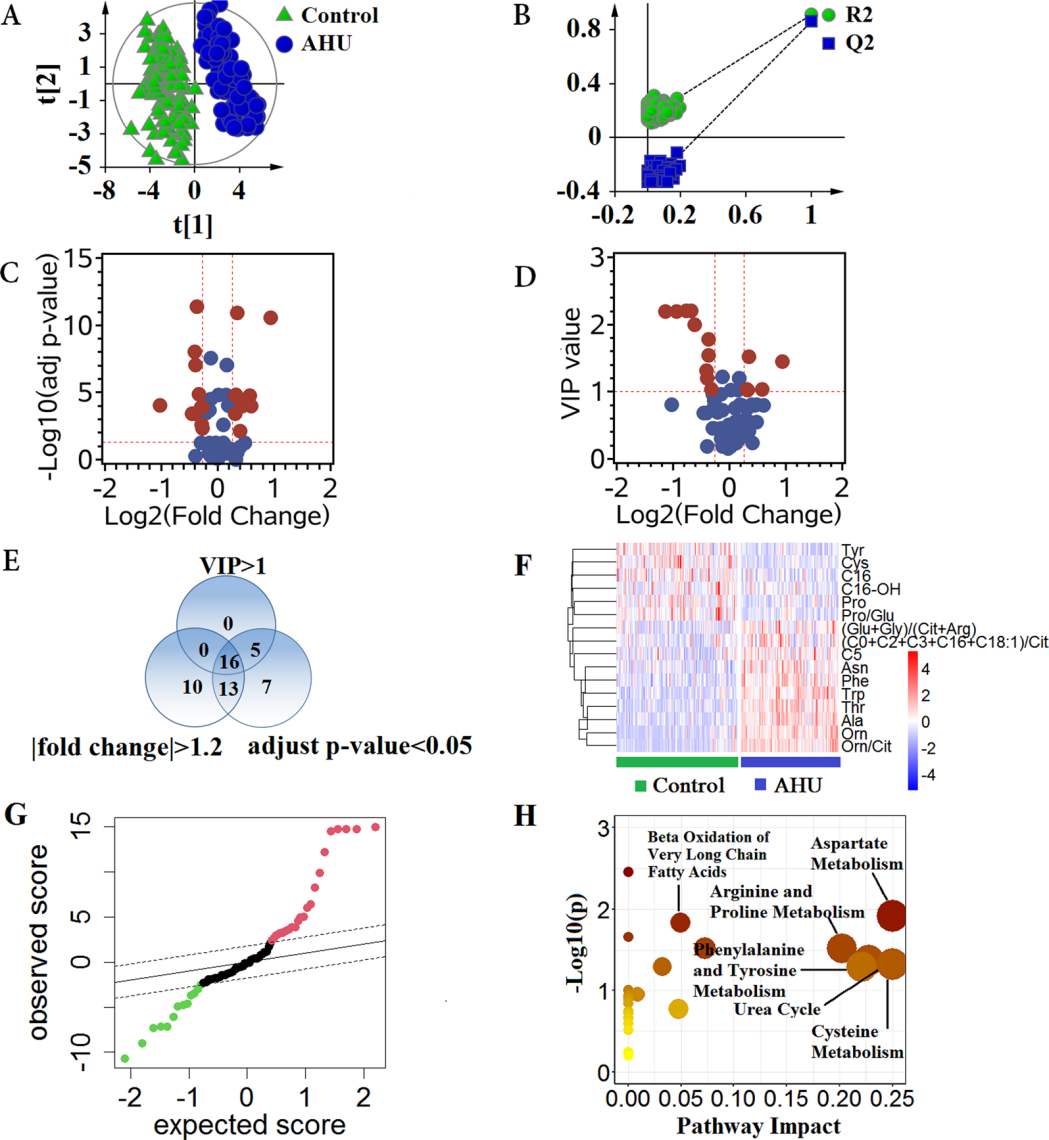
Metabolic profiles distinguish patients with AHU from healthy controls. **A** Score plot of PLS-DA analysis. **B** A 200-times permutation test for assessing the performance of PLS-DA model. The y-axis intercepts in test plot were R2 = (0.0, 0.154) and Q2 = (0.0, −0.368). **C** The volcano plot of adjusted *p*-value versus fold change (FC). Significantly altered metabolites were defined with adjusted *p*-value < 0.05 and FC > 1.2 or < −1.2. The selected metabolites were colored in red. **D** The volcano plot of VIP versus FC. Metabolites with VIP > 1 and FC > 1.2 or < −1.2 were selected. **E** Venn diagram showed the differential metabolites between patients with AHU and healthy individuals. Sixteen metabolites were screened with adjusted *p*-value < 0.05 and VIP > 1 and FC > 1.2 or < −1.2. **F** Heatmap of the 16 selected metabolites. Red indicates up-regulated metabolites, and blue indicates down-regulated metabolites in patients with AHU compared with healthy individuals. **G** Significance analysis of microarrays in patients with AHU and healthy individuals. The levels of 13 metabolites were down-regulated, and 25 metabolites were up-regulated in patients with AHU compared with healthy individuals. **H** Pathway enrichment plot based on differential metabolites between the two groups. AHU, asymptomatic hyperuricemia

The PLS-DA method was performed to supervise the separation between AHU and control group, as shown in Fig. 3A. PLS-DA score plot showed a better separation for all the detected metabolites between AHU and control group without over-fitting (Fig. 3B), and important variables contributing to this classification could be selected based on VIP value. As shown in Fig. 3C and D, according to calculated FC, univariate statistical test, and PLS-DA between the two groups, 16 metabolites were selected with FC > 1.2 or < − 1.2, adjusted *p*-value < 0.05, and VIP > 1 (Fig. 3E). Heatmap cluster analysis showed a clear separation between the two groups (Fig. 3F). Among these selected metabolites, 10 metabolites were significantly up-regulated, conversely, 5 metabolites were distinctively down-regulated in patients with AHU compared with healthy individuals based on SAM analysis (Fig. 3G). Pathway enrichment analysis demonstrated that these significantly altered metabolites were primarily related to 5 pathways: aspartate metabolism, arginine and proline metabolism, phenylalanine and tyrosine metabolism, urea cycle, cysteine metabolism, and β-oxidation of very long chain fatty acids (Fig. 3H).

### Comparison of metabolic profiles between patients with gout and control group

**Fig. 4:**
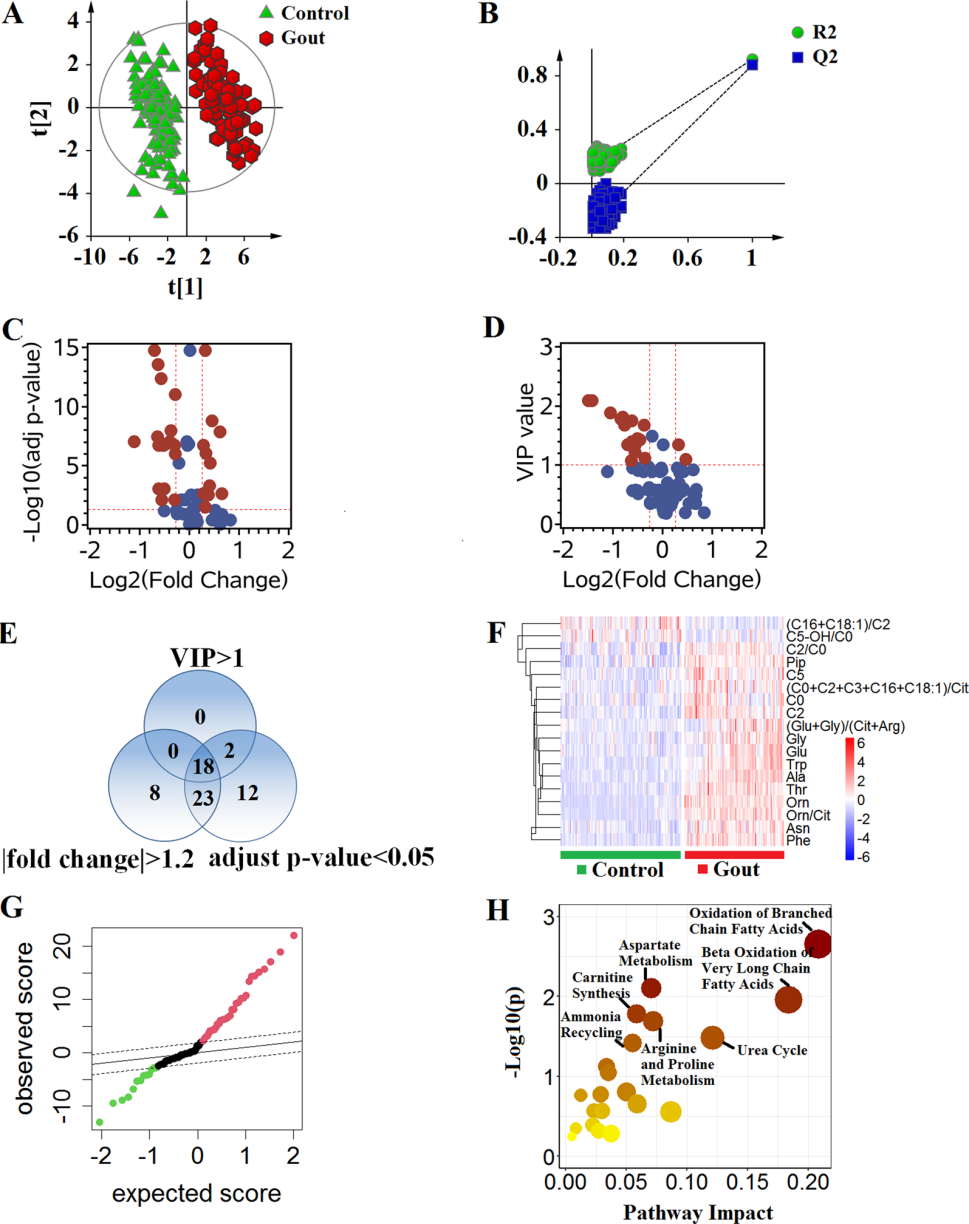
Metabolic profiles distinguish patients with gout from healthy individuals. **A** Score plot of PLS-DA analysis. **B** A 200-times permutation test for assessing the performance of PLS-DA model. The y-axis intercepts in test plot were R2 = (0.0, 0.144) and Q2 = (0.0, −0.297). **C** The volcano plot of adjusted *p*-value versus fold change (FC). Significantly altered metabolites were defined with adjusted *p*-value < 0.05 and FC > 1.2 or < −1.2. The selected metabolites were colored in red. **D** The volcano plot of VIP versus FC. Metabolites with VIP > 1 and FC > 1.2 or < −1.2 were selected. **E** Venn diagram showed the differential metabolites between patients with gout and healthy individuals. Eighteen metabolites were screened with adjusted *p*-value < 0.05 and VIP > 1 and FC > 1.2 or < −1.2. **F** Heatmap of the 18 selected metabolites. Red indicates up-regulated metabolites, and blue indicates down-regulated metabolites in patients with gout compared with healthy individuals. **G** Significance analysis of microarrays in patients with gout and healthy individuals. The levels of 13 metabolites were down-regulated, and 33 metabolites were up-regulated in patients with gout compared with healthy individuals. **H** Pathway enrichment plot based on differential metabolites between the two groups

We performed PLS-DA classification to compare the metabolic profiles for gout and control group. There is an obvious overall metabolic separation between the two groups without over-fitting (Fig. 4A and B). As shown in Fig. 4C–E, 18 metabolites with FC > 1.2 or < − 1.2, adjusted *p*-value < 0.05 and VIP > 1 were significantly altered in gout compared with control group. The heatmap showed that significantly changed metabolites clustered in the two groups (Fig. 4F). In these metabolites, 16 metabolites were significantly up-regulated, and 1 metabolite was distinctly down-regulated in patients with gout according to SAM analysis (Fig. 4G). We found that oxidation of branched chain fatty acids, β-oxidation of very long chain fatty acids, urea cycle, aspartate metabolism, carnitine synthesis, ammonia recycling, and arginine and proline metabolism pathways were significantly perturbed in the two groups (Fig. 4H).

### Comparison of metabolic profiles between gout and AHU

**Fig. 5:**
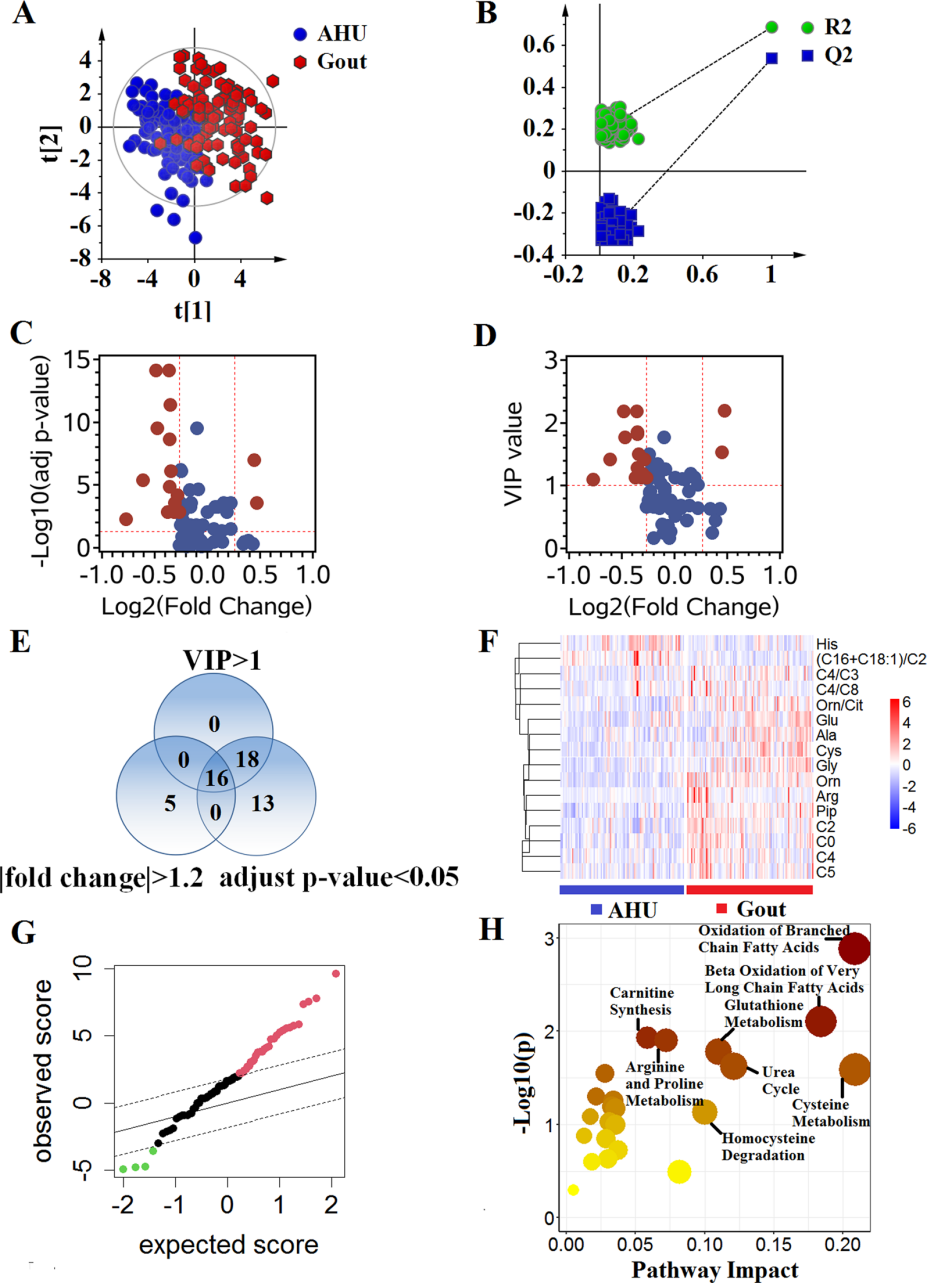
Metabolic profiles distinguishing patients with gout from patients with AHU. **A** Score plot of PLS-DA analysis. **B** A 200-times permutation test for assessing the performance of PLS-DA model. The y-axis intercepts in test plot were R2 = (0.0, 0.182) and Q2 = (0.0, −0.343). **C** The plot of adjusted *p*-value versus fold change (FC). Significantly altered metabolites were defined with adjusted *p*-value < 0.05 and FC > 1.2 or < −1.2. The selected metabolites were colored in red. **D** The plot of VIP versus FC. Metabolites with VIP > 1 and FC > 1.2 or < −1.2 were selected. **E** Venn diagram showed the differential metabolites between patients with gout and patients with AHU. Sixteen metabolites were screened with adjusted *p*-value < 0.05 and VIP > 1 and FC > 1.2 or < −1.2. **F** Heatmap of different metabolites. Red indicates up-regulated metabolites, and blue indicates down-regulated metabolites in patients with gout compared with AHU. **G** Significance analysis of microarrays in patients with gout and patients with AHU. The levels of 4 metabolites were down-regulated, and 30 metabolites were up-regulated in patients with gout compared with patients with AHU. **H** Pathway enrichment plot based on differential metabolites between the two groups. AHU, asymptomatic hyperuricemia

The PLS-DA score plot showed a clear overall separation between gout and AHU, as shown in Fig. 5A and B, which indicated the distinct metabolic profiles in the two groups. Volcano plots were used to further select significantly altered metabolites. As shown in Fig. 5C and D, 16 metabolites (Fig. 5E) with FC > 1.2 or < − 1.2, adjusted *p*-value < 0.05 and VIP > 1 showed distinct change in gout compared with AHU. Simultaneously, all the selected metabolites exhibited a clear cluster in heatmap (Fig. 5F). In the 16 metabolites, 13 metabolites were obviously up-regulated, and 2 metabolites were significantly down-regulated in gout compared with AHU (Fig. 5G). There were 6 pathways, including oxidation of branched chain fatty acids, β-oxidation of very long chain fatty acids, glutathione metabolism, urea cycle, cysteine metabolism, homocysteine degradation, carnitine synthesis, and arginine and proline metabolism were significantly altered between the two groups (Fig. 5H).

### Selection of important metabolite

**Fig. 6:**
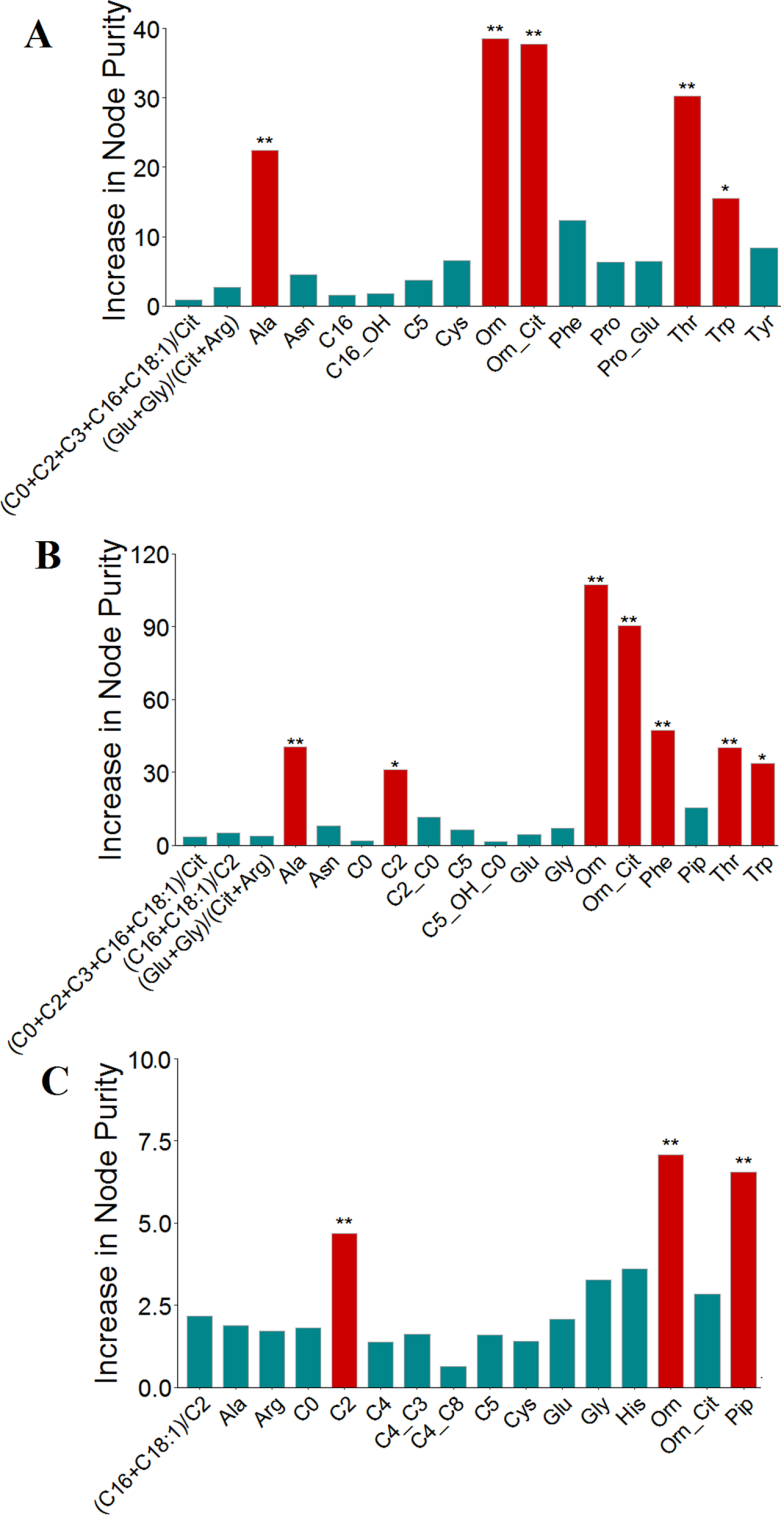
The identification of important metabolites. The increase in node purity was used to rank the relative importance of metabolites in random forest model. **A** The contribution of metabolites in random forest model for AHU and control group. **B** The contribution of metabolites in random forest model for gout and control group. **C** The contribution of metabolites in random forest model for AHU and gout group. The higher increase in mode purity values indicated more important features. Levels of statistical significance are as follows: *p < 0.05 and **p < 0.01. Orn, Ornithine; His, Histidine; Pip, Piperamide; C2, Acetylcarnitine; Gly, Glycine; C4, Butyrylcarnitine; C16, Palmitoylcarnitine; Ala, Alanine; Arg, Arginine; Cit, Citrulline; Glu, Glutamic; Cys, Cysteine; C5, Isovalerylcarnitine; C3, Propionylcarnitine; C0, Free carnitine

After systematical selection, the random forest algorithm was adopted to rank the importance of above selected metabolites, and important metabolites with *p*-value < 0.05 were retained by 5000 permutations of response variable. A total of 5 metabolites were screened based on the importance of differential variables in AHU and control group by using random forest algorithm, as shown in Fig. 6A. Orn was observed to be the most important metabolite for predicting AHU. Other important variables for predicting AHU were Orn/Cit, Thr, Ala, and Trp. In order to distinguish gout patients from healthy individuals, 7 important metabolites were screened based on the significance of each features on response variable. As displayed in Fig. 6B, Orn is the most important feature for predicting gout. Other important features were Orn/Cit, Phe, Ala, Phe, Thr, Trp, and C2. For distinguishing gout patients from AHU patients, 3 important metabolites were obtained by random forest analysis. Orn was found to be the most important metabolite for distinguishing gout from AHU. Pip and C2 also contributed to this classification, as shown in Fig. 6C.

## Discussion

Gout has become to a public health problem worldwide. In the last few decades, the prevalence of gout has risen rapidly. It affects patients’ quality of life, and causes excruciating painful acute attacks of arthritis. Gout has complex pathogenesis. AHU has been regarded as a risk factor to gout progression [27]. Many patients with AHU have monosodium urate crystals and occur acute attacks of gout, however, several of them do not have this trend [6]. If use SUA as a biomedical indicator, the diagnosis of gout may be inaccurate, even though the crystal was detected in digit joint [28]. Hence, developing novel biomarkers beyond serum urate levels related to the occurrence and development of gout may contribute to promote diagnosis, refine stages of gout, and improve care in comorbidities of hyperuricemia and gout [29]. Furthermore, AHU and gout are metabolic diseases, and metabolomics method can be applied to discover biomarkers and depict metabolism changes in biological systems for patients with AHU or gout, which is desirable to prevent acute attacks of gout and destruction of joints.

We carried out metabolomics study based on a combination of DBS sampling and MS technology to systematically define metabolic profiles for patients with AHU and gout toward detected amino acids, carnitine/acylarinieint, and their ratios. After systematical selection, panels of metabolites were screened to differentiate control, AHU, and gout groups.

The important underlying mechanism of gout was referred to the increased levels of UA in the blood. Persistent increase of UA levels triggers crystallization of UA and deposition of monosodium urate crystals, which can cause painful attacks of acute gout [30]. The SUA is the final product of purine metabolism [31]. Some amino acids were involved in the biosynthesis of purine and formed UA. Accumulated evidence links various amino acid levels to AHU and gout. In this study, metabolomic profile analysis showed that several amino acids were significantly altered among controls, AHU, and gout. Levels of Orn, Orn/Cit, Thr, Ala, Trp, Phe, Asn, C5, (Glu + Gly)/(Cit + Arg) were significantly higher in both AHU and gout groups compared with control group in this study. A previous study showed that concentrations of Trp, Phe, and Ala were significantly up-regulated in gout compared with controls [32], which was consistent with our analysis results. However, our study also reported significantly increased levels of Pro, Leu, and Val, as well as decreased levels of Ser and Gly, which were inconsistent with our observations. Several factors may generate the contradictory findings. There were different genetic background and lifestyle for the participants and different sample size in the two studies. In addition, in the previously reported study, the patients were not defined as acute gout attack. Furthermore, there were significant differences of gender and BMI for the participants in the previous study. It has been found that the levels of Thr, Trp, and Ala were increased in patients with HUA compared with healthy individuals [33], which were consistent with our findings. It is worth noting that abnormalities in tryptophan metabolism showed association with AHU [34] and acute gout [35]. The altered levels of Trp may be owing to the UA-induced dysfunction of multidrug resistance protein 4 (MRP4) and breast cancer resistance protein (BCRP) [36]. Amino acids are also the essential nutrients as energy source in human body. Ala is a precursor for gluconeogenesis in the liver. It can be synthesized from branched chain amino acid (BCAA) under starvation condition, and then transported to liver for making glucose to meet energy demands [37]. The increased levels of Ala indicated that energy supply in patients with AHU or gout was disturbed and most patients may suffered unmet energy demand. Besides, Tyr as a ketogenic and glycogenic amino acid and Pro as a glycogenic amino acid in human body, all of them are vital to generate energy in human body. It has been proved that the degradation of pro was closely associated with TCA cycle and urea cycle [38]. The decreased levels of Tyr and Pro indicated the increased body’s energy consumption in patients with AHU. Gly, Glu, and Cys acts as the components of glutathione, the primary antioxidant in the body, which could protect cells from reactive oxygen species and free radicals and maintain cellular homeostasis via the regulation of oxidative stress and detoxification [39]. Oxidative stress may affect the development and manifestation for patients with gout [40]. In our study, accumulated Gly, Glu, and Cys in gout suggested the dysfunction in glutathione metabolism, which indicated that abnormal energy conversion and antioxidant capacity may be more severe in gout patients than in AHU patients. The levels of Orn and Orn/Cit ratio were up-regulated in AHU and gout compared with control group, and Arg levels were only increased in gout compared with AHU. Orn, Arg, and Cit take part in the urea metabolism, and urea cycle disorders could influence oxidative stress [41]. A previous study showed distinct serum metabolomic signatures between AHU and gout, and arginine metabolism seems to play a crucial role [18], which were consistent with our finding from pathway analysis. Another study showed that arginine anabolic metabolism can dedicate to the progression of inflammation in patients with gout [42], and it could be used as a biomarker to distinguish between hyperuricemia and gout [18]. The up-regulated Orn levels and Orn/Cit ratio indicated the disordered urea cycle in AHU and gout, which subsequently influence oxidation stress. Besides, up-regulated levels of Arg in patients with gout may be suggested inflammation progression in gout. The (Glu + Gly)/(Cit + Arg) ratio was appropriate to discriminate heterozygous female patients with ornithine transcarbamylase deficiency (OTCD) from others, and this disease is the most common urea cycle disorder [43]. The increased (Glu + Gly)/(Cit + Arg) ratio in both AHU and gout may further imply the disordered urea cycle in AHU and gout. Histidine is an important material in the biosynthesis of purine via de novo synthesis pathway which needs a lot of energy [44]. The decreased His levels were only seen in gout patients, which indicated the acceleration of purine metabolism and excessive energy depletion in gout. Overall, these findings suggested a link between patients with AHU or gout and disordered amino acid metabolism, which could provide a new direction for preventing acute attacks of gout.

In order to guarantee continuous energy supply in human body, considerable amounts of fat besides glucose was oxidized. In this process, carnitine/acylcarnitine transporter activates long-chain fatty acids from cytosol into mitochondrion, which is essential for fatty acid oxidation and energy metabolism. Deficiency of carnitine/acylcarnitine may influence the utilization of fat as fuel [45]. In addition, it has been observed that levels of long-chain acylcarnitines were associated with incident hyperuricemia [23]. In this study, we only found one long-chain acylcarnitine (C16) was significantly down-regulated in patients with AHU compared with healthy individuals, which implied the impaired fatty acid β-oxidation in AHU. Significantly altered (C16 + C18:1)/C2 ratio was only observed in patients with gout. This ratio has been used to improve the diagnostic specificity for carnitine-acylcarnitine translocase (CACT) deficiency and carnitine palmitoyl transferase 2 (CPT2) deficiency, which were regarded as inherited disorders of mitochondrial long-chain fatty acid oxidation [46]. In this study, the increased (C16 + C18:1)/C2 ratio may be influenced by the disordered mitochondrial long-chain fatty acid oxidation in patients with gout. Besides long-chain acylcarnitines, several short-chain carnitines were accumulated in AHU and gout. C5 levels were significantly increased in AHU and gout compared with control group. Furthermore, obviously up-regulated levels of C0 and C2 were only shown in patients with gout. Moreover, increased levels of C4 can contribute to increased C4/C3 ratio in patients with gout compared with patients with AHU. As an important substance in energy metabolism in the cells, significantly increased carnitine levels were probably related to an active energy metabolism in AHU and gout [47]. It is widely known that lipotoxicity was closely associated with development of gout [48, 49]. The accumulation of acylcarnitines may indicate the lipotoxicity-induced mitochondrial stress in AHU and gout [50]. Based on these findings, altered metabolism of carnitine/acylcarnitine was observed in AHU, especially in patients with gout who have more changed carnitine/acylcarnitine. Our findings might shed light on discovering new biomarkers for AHU and gout and further preventing and controlling related diseases.

There are several limitations in the present study. Firstly, owing to the trade-off between coverage and cost, the number of detected metabolites was limited in this study, and more metabolites such as fatty acids will be collected for observing more potential biomarkers; Secondly, a multi-institution study with a larger sample size is required to further validate this study’s findings; Thirdly, it is warranted to conduct a longitudinal study for AHU and gout to further validate our findings. Gout can process through four pathophysiological stages: hyperuricemia without MSU crystal deposition, MSU crystals without signs of gout, acute gout flares, advanced gout, therefore, this study will be more systematic when a reasonable amount of patients at interval stage of gout are recruited; Fourthly, for the limited number of females in AHU and gout groups, more females will be collected and can be hierarchically analyzed according to menopausal age; Fifthly, the patients recruited in this study without common comorbidities related to gout may restrict generalizability to general gout population; Finally, although age, gender, and BMI were matched among control, AHU, gout groups, some other confounding factors such as dietary habit are needed to evaluate our results.

## Conclusion

To sum up, a combination of DBS sampling and MS technology was applied to detect metabolic changes in AHU and gout. Significant dysregulation of metabolic pathway was observed in AHU and gout patients. Important altered metabolites, including Orn, Pip, and C2, were screened for distinguishing gout patients from AHU patients by random forest analysis. Our results may be helpful in detecting patients with AHU who are at a high risk of gout. Such a novel method may have profound effects on early prediction and diagnosis of gout.

### Supplementary Information

**Additional file 1: Figure S1. **A 200-times permutation test for assessing the performance of PLS-DA model among control group, asymptomatic hyperuricemia, and gout. **Table S1.** Detected metabolites in control, AHU, and gout groups.

## Data Availability

The original contributions presented in the study are included in the article/ Additional file; further inquiries can be directed to the corresponding authors.

## Abbreviations

AHU: Asymptomatic hyperuricemia
MS: Mass spectrometry
DBS: Dried blood spot
Orn: Ornithine
His: Histidine
Pip: Piperamide
C0: Free carnitine
C2: Acetylcarnitine
C3: Propionylcarnitine
Gly: Glycine
C4: Butyrylcarnitine
C16: Palmitoylcarnitine
MSU: Monosodium urate
SUA: Serum uric acid
GC: Gas chromatography
LC: Liquid chromatography
AGA: Acute gout arthritis
QC: Quality control
PCA: Principal component analysis
PLS-DA: Partial least squares discriminant analysis
VIP: Variable importance in projection
FDR: False discovery rate
ROC: Receiver-operating characteristic
AUC: Area under the ROC curve
BMI: Body-Mass Index
LDL: Low density lipoprotein
SBP: Systolic blood pressure
DBP: Diastolic blood pressure
AST: Aspartate aminotransferase
ALT: Alanine aminotransferase
HDL: High density lipoprotein
eGFR: Estimated glomerular filtration rate
Thr: Threonine
Trp: Tryptophan
Pro: Proline
Tyr: Tyrosine
C5: Isovalerylcarnitine
Arg: Arginine
Cit: Citrulline
Ala: Alanine
Phe: Phenylalanine
Asn: Asparagine
Glu: Glutamic
Val: Glutamic
Leu: Leucine
Ser: Serine
MRP4: Multidrug resistance protein 4
BCRP: Breast cancer resistance protein
BCAA: Branched chain amino acid
Cys: Cysteine
OTCD: Ornithine transcarbamylase deficiency
CACT: Carnitine-acylcarnitine translocase

## References

[1] Towiwat P, Chhana A, Dalbeth N (2019). The anatomical pathology of gout: a systematic literature review. BMC Musculoskelet Disord.

[2] Dehlin M, Jacobsson L, Roddy E (2020). Global epidemiology of gout: prevalence, incidence, treatment patterns and risk factors. Nat Rev Rheumatol.

[3] Xiaofeng Z, Yaolong C (2017). 2016 China guidelines for management of gout. Chin J Intern Med.

[4] Dalbeth N, Stamp L (2014). Hyperuricaemia and gout: time for a new staging system?. Ann Rheum Dis.

[5] Dalbeth N, House ME, Aati O, Tan P, Franklin C, Horne A (2015). Urate crystal deposition in asymptomatic hyperuricaemia and symptomatic gout: a dual energy CT study. Ann Rheum Dis.

[6] Zhang WZ (2021). Why does hyperuricemia not necessarily induce gout?. Biomolecules.

[7] Soltani Z, Rasheed K, Kapusta DR, Reisin E (2013). Potential role of uric acid in metabolic syndrome, hypertension, kidney injury, and cardiovascular diseases: is it time for reappraisal?. Curr Hypertens Rep.

[8] Puig JG, Martínez MA (2008). Hyperuricemia, gout and the metabolic syndrome. Curr Opin Rheumatol.

[9] Bardin T, Richette P (2017). Impact of comorbidities on gout and hyperuricaemia: an update on prevalence and treatment options. BMC Med.

[10] Souto-Carneiro M, Tóth L, Behnisch R, Urbach K, Klika KD, Carvalho RA (2020). Differences in the serum metabolome and lipidome identify potential biomarkers for seronegative rheumatoid arthritis versus psoriatic arthritis. Ann Rheum Dis.

[11] Holers VM (2021). Challenges and opportunities: using omics to generate testable insights into pathogenic mechanisms in preclinical seropositive rheumatoid arthritis. Arthritis Rheumatol.

[12] Kaushik AK, DeBerardinis RJ (2018). Applications of metabolomics to study cancer metabolism. Biochim Biophys Acta Rev Cancer.

[13] Simonian M, Mosallayi M, Mirzaei H (2018). Circulating miR-21 as novel biomarker in gastric cancer: diagnostic and prognostic biomarker. J Cancer Res Ther.

[14] Bujak R, Struck-Lewicka W, Markuszewski MJ, Kaliszan R (2015). Metabolomics for laboratory diagnostics. J Pharm Biomed Anal.

[15] Chandran V, Rahman P (2020). Predicting therapeutic response through biomarker analysis in psoriatic arthritis, an example of precision medicine. Expert Rev Precis Med Drug Dev.

[16] Albrecht E, Waldenberger M, Krumsiek J, Evans AM, Jeratsch U, Breier M (2014). Metabolite profiling reveals new insights into the regulation of serum urate in humans. Metabolomics.

[17] Wang W, Kou J, Zhang M, Wang T, Li W, Wang Y (2023). A metabonomic study to explore potential markers of asymptomatic hyperuricemia and acute gouty arthritis. J Orthop Surg Res.

[18] Shen X, Wang C, Liang N, Liu Z, Li X, Zhu ZJ (2021). Serum metabolomics identifies dysregulated pathways and potential metabolic biomarkers for hyperuricemia and gout. Arthritis Rheumatol.

[19] Guo X, Zhou L, Wang Y, Suo F, Wang C, Zhou W (2023). Development of a fast and robust liquid chromatography-mass spectrometry-based metabolomics analysis method for neonatal dried blood spots. J Pharm Biomed Anal.

[20] Zhao G, Cheng D, Wang Y, Cao Y, Xiang S, Yu Q (2020). A metabolomic study for chronic heart failure patients based on a dried blood spot mass spectrometry approach. RSC Adv.

[21] Yu L, Li K, Li X, Guan C, Sun T, Zhang X (2020). Metabolomic profiling of dried blood spots reveals gender-specific discriminant models for the diagnosis of small cell lung cancer. Aging.

[22] Kelley WN, Holmes EW, Van der Weyden MB (1975). Current concepts on the regulation of purine biosynthesis de novo in man. Arthritis Rheum.

[23] Wang F, Sun L, Zong G, Gao X, Zhang H, Xiong Q (2020). Associations of amino acid and acylcarnitine profiles with incident hyperuricemia in middle-aged and older chinese individuals. Arthritis Care Res.

[24] Neogi T, Jansen TL, Dalbeth N, Fransen J, Schumacher HR, Berendsen D (2015). 2015 Gout classification criteria: an American College of Rheumatology/European League Against Rheumatism collaborative initiative. Ann Rheum Dis.

[25] Wang Q, Sun T, Cao Y, Gao P, Dong J, Fang Y (2016). A dried blood spot mass spectrometry metabolomic approach for rapid breast cancer detection. Onco Targets Ther.

[26] Benjamini Y, Hochberg Y (1995). Controlling the false discovery rate—a new and powerful approach to multiple testing. J R Stat Soc B.

[27] Singh JA, Reddy SG, Kundukulam J (2011). Risk factors for gout and prevention: a systematic review of the literature. Curr Opin Rheumatol.

[28] Guggi V, Calame L, Gerster JC (2002). Contribution of digit joint aspiration to the diagnosis of rheumatic diseases. Joint Bone Spine.

[29] Dalbeth N, Choi HK, Terkeltaub R (2017). Review: gout: a roadmap to approaches for improving global outcomes. Arthritis Rheumatol.

[30] Scott JT (1978). New knowledge of the pathogenesis of gout. J Clin Pathol Suppl (R Coll Pathol).

[31] Maiuolo J, Oppedisano F, Gratteri S, Muscoli C, Mollace V (2016). Regulation of uric acid metabolism and excretion. Int J Cardiol.

[32] Mahbub MH, Yamaguchi N, Takahashi H, Hase R, Amano H, Kobayashi-Miura M (2017). Alteration in plasma free amino acid levels and its association with gout. Environ Health Prev Med.

[33] Luo Y, Wang L, Liu XY, Chen X, Song YX, Li XH (2018). Plasma profiling of amino acids distinguishes acute gout from asymptomatic hyperuricemia. Amino Acids.

[34] Kong X, Liang H, An W, Bai S, Miao Y, Qiang J (2023). Rapid identification of early renal damage in asymptomatic hyperuricemia patients based on urine Raman spectroscopy and bioinformatics analysis. Front Chem.

[35] Liu Y, Sun X, Di D, Quan J, Zhang J, Yang X (2011). A metabolic profiling analysis of symptomatic gout in human serum and urine using high performance liquid chromatography-diode array detector technique. Clin Chim Acta.

[36] Dankers AC, Mutsaers HA, Dijkman HB, van den Heuvel LP, Hoenderop JG, Sweep FC (2013). Hyperuricemia influences tryptophan metabolism via inhibition of multidrug resistance protein 4 (MRP4) and breast cancer resistance protein (BCRP). Biochim Biophys Acta.

[37] Felig P (1973). The glucose-alanine cycle. Metabolism.

[38] Pandhare J, Donald SP, Cooper SK, Phang JM (2009). Regulation and function of proline oxidase under nutrient stress. J Cell Biochem.

[39] Ribas V, García-Ruiz C, Fernández-Checa JC (2014). Glutathione and mitochondria. Front Pharmacol.

[40] Alduraibi FK, Saleem M, Ricart K, Patel RP, Szalai AJ, Singh JA (2023). Clustering patients with gout based on comorbidities and biomarkers: a cross-sectional study. J Rheumatol.

[41] Lopes FF, Lamberty Faverzani J, Hammerschmidt T, Aguilar Delgado C, Ferreira de Oliveira J, Wajner M (2023). Evaluation of oxidative damage to biomolecules and inflammation in patients with urea cycle disorders. Arch Biochem Biophys.

[42] Zhang Y, Zhang H, Chang D, Guo F, Pan H, Yang Y (2018). Metabolomics approach by 1H NMR spectroscopy of serum reveals progression axes for asymptomatic hyperuricemia and gout. Arthritis Res Ther.

[43] Iijima H, Kubota M (2022). A simple screening method for heterozygous female patients with ornithine transcarbamylase deficiency. Mol Genet Metab.

[44] Boza JJ, Moënnoz D, Bournot CE, Blum S, Zbinden I, Finot PA (2000). Role of glutamine on the de novo purine nucleotide synthesis in Caco-2 cells. Eur J Nutr.

[45] Xu Y, Jiang W, Chen G, Zhu W, Ding W, Ge Z (2017). L-carnitine treatment of insulin resistance: a systematic review and meta-analysis. Adv Clin Exp Med.

[46] Habib A, Azize NAA, Rahman SA, Yakob Y, Suberamaniam V, Nazri MIBA (2021). Novel mutations associated with carnitine-acylcarnitine translocase and carnitine palmitoyl transferase 2 deficiencies in Malaysia. Clin Biochem.

[47] You L, Zheng F, Su C, Wang L, Li X, Chen Q (2022). Metabolome-wide association study of serum exogenous chemical residues in a cohort with 5 major chronic diseases. Environ Int.

[48] Joosten LA, Netea MG, Mylona E, Koenders MI, Malireddi RK, Oosting M (2010). Engagement of fatty acids with Toll-like receptor 2 drives interleukin-1β production via the ASC/caspase 1 pathway in monosodium urate monohydrate crystal-induced gouty arthritis. Arthritis Rheum.

[49] Li C, Hsieh MC, Chang SJ (2013). Metabolic syndrome, diabetes, and hyperuricemia. Curr Opin Rheumatol.

[50] Schooneman MG, Vaz FM, Houten SM, Soeters MR (2013). Acylcarnitines: reflecting or inflicting insulin resistance?. Diabetes.

