# Supplementary material for: Metabolomic analysis for asymptomatic hyperuricemia and gout based on a combination of dried blood spot sampling and mass spectrometry technology
Source: J Orthop Surg Res. 2023 Oct 11;18:769. doi: 10.1186/s13018-023-04240-3 (PMC10566066; doi:10.1186/s13018-023-04240-3)
Supplement: Supplementary file 1 — Additional file 1: Figure S1. A 200-times permutation test for assessing the performance of PLS-DA model among control group, asymptomatic hyperuricemia, and gout. Table S1. Detected metabolites in control, AHU, and gout groups. [file 13018_2023_4240_MOESM1_ESM.docx]

**Supplementary Material**


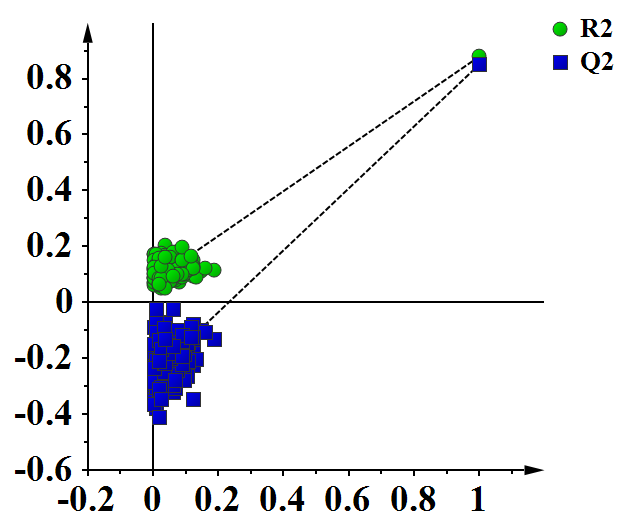


Figure S1 A 200-times permutation test for assessing the performance of PLS-DA model among control group, asymptomatic hyperuricemia, and gout. The y-axis intercepts in test plot were R2=(0.0, 0.0769) and Q2=(0.0,-0.257).

Table S1 Detected metabolites in control, AHU, and gout groups

| **Metabolites** | **Control(N=114)** |  | **AHU(N=92)** |  | **Gout(N=94)** |
| --- | --- | --- | --- | --- | --- |
| Ala | 190.405±56.204 |  | 324.881±75.067 |  | 395.450±112.208 |
| Arg | 7.359±4.829 |  | 6.105±2.579 |  | 10.404±9.948 |
| Asn | 79.665±18.639 |  | 103.053±22.311 |  | 109.767±24.110 |
| Cit | 25.014±7.065 |  | 21.509±6.252 |  | 23.732±7.424 |
| Cys | 1.451±0.828 |  | 0.758±0.378 |  | 1.158±0.591 |
| Gln | 9.427±3.941 |  | 8.330±4.234 |  | 7.693±4.919 |
| Glu | 148.854±45.635 |  | 179.151±67.781 |  | 229.054±74.809 |
| Gly | 184.971±44.921 |  | 205.034±35.428 |  | 261.996±63.440 |
| Hcy | 8.782±0.949 |  | 9.554±0.860 |  | 10.152±0.849 |
| His | 73.202±43.077 |  | 66.170±30.652 |  | 47.692±19.183 |
| Leu | 119.129±26.921 |  | 123.363±25.536 |  | 137.499±29.177 |
| Lys | 154.596±71.135 |  | 159.588±81.116 |  | 147.381±94.242 |
| Orn | 15.274±7.142 |  | 29.278±6.558 |  | 40.942±9.614 |
| Phe | 39.333±7.807 |  | 50.831±8.966 |  | 60.189±12.818 |
| Pip | 245.037±100.964 |  | 283.145±60.771 |  | 360.780±74.172 |
| Pro | 489.634±153.592 |  | 395.813±116.740 |  | 470.580±149.267 |
| Ser | 49.519±14.842 |  | 53.826±6.686 |  | 60.250±9.633 |
| Thr | 26.136±6.907 |  | 41.842±8.409 |  | 45.748±10.957 |
| Trp | 46.330±12.942 |  | 71.304±16.554 |  | 82.793±21.379 |
| Tyr | 56.287±13.838 |  | 44.229±9.020 |  | 52.551±11.043 |
| Val | 155.608±33.320 |  | 145.467±27.992 |  | 155.182±32.016 |
| C0 | 32.519±8.780 |  | 32.979±9.493 |  | 41.801±12.073 |
| C2 | 12.535±3.648 |  | 15.390±5.304 |  | 21.335±5.745 |
| C3 | 1.813±0.585 |  | 1.745±0.889 |  | 1.789±0.812 |
| C4 | 0.208±0.082 |  | 0.231±0.090 |  | 0.299±0.136 |
| C4-OH | 0.044±0.022 |  | 0.053±0.028 |  | 0.062±0.024 |
| C4DC | 0.367±0.217 |  | 0.278±0.085 |  | 0.326±0.114 |
| C5 | 0.122±0.047 |  | 0.160±0.046 |  | 0.198±0.071 |
| C5-OH | 0.221±0.102 |  | 0.196±0.076 |  | 0.218±0.067 |
| C5DC | 0.099±0.052 |  | 0.102±0.039 |  | 0.121±0.055 |
| C8 | 0.109±0.065 |  | 0.121±0.087 |  | 0.130±0.086 |
| C10 | 0.124±0.081 |  | 0.170±0.127 |  | 0.177±0.142 |
| C12 | 0.071±0.031 |  | 0.063±0.029 |  | 0.065±0.028 |
| C14 | 0.065±0.026 |  | 0.069±0.024 |  | 0.072±0.027 |
| C14-OH | 0.026±0.015 |  | 0.021±0.009 |  | 0.025±0.010 |
| C14DC | 0.022±0.009 |  | 0.018±0.008 |  | 0.020±0.009 |
| C14:1 | 0.076±0.041 |  | 0.064±0.027 |  | 0.077±0.035 |
| C16 | 0.941±0.305 |  | 0.754±0.240 |  | 0.825±0.339 |
| C16-OH | 0.024±0.012 |  | 0.019±0.009 |  | 0.018±0.007 |
| C16:1-OH | 0.042±0.018 |  | 0.038±0.014 |  | 0.041±0.019 |
| C18 | 0.547±0.209 |  | 0.654±0.197 |  | 0.717±0.237 |
| C20 | 0.025±0.021 |  | 0.020±0.007 |  | 0.023±0.008 |
| C22 | 0.044±0.022 |  | 0.043±0.015 |  | 0.043±0.013 |
| C24 | 0.035±0.024 |  | 0.025±0.010 |  | 0.026±0.009 |
| Cit/Arg | 5.404±4.136 |  | 3.991±1.727 |  | 3.422±1.842 |
| Orn/Cit | 0.652±0.343 |  | 1.429±0.385 |  | 1.836±0.538 |
| Val/Phe | 4.039±0.924 |  | 2.892±0.471 |  | 2.629±0.514 |
| C2/C0 | 0.406±0.132 |  | 0.490±0.165 |  | 0.525±0.108 |
| C3/C0 | 0.059±0.022 |  | 0.054±0.022 |  | 0.043±0.014 |
| C3/C2 | 0.151±0.048 |  | 0.114±0.039 |  | 0.085±0.031 |
| C4/C2 | 0.017±0.007 |  | 0.016±0.008 |  | 0.014±0.006 |
| C4/C3 | 0.120±0.041 |  | 0.147±0.058 |  | 0.183±0.083 |
| C4/C8 | 2.440±1.425 |  | 2.488±1.753 |  | 2.993±2.194 |
| C5-OH/C0 | 0.007±0.003 |  | 0.006±0.002 |  | 0.006±0.002 |
| C5DC/C5-OH | 0.526±0.367 |  | 0.572±0.248 |  | 0.588±0.276 |
| C5DC/C16 | 0.114±0.070 |  | 0.150±0.077 |  | 0.167±0.093 |
| C8/C2 | 0.009±0.006 |  | 0.008±0.005 |  | 0.006±0.004 |
| C16-OH/C16 | 0.027±0.016 |  | 0.027±0.014 |  | 0.025±0.013 |
| C14:1/C16 | 0.087±0.051 |  | 0.093±0.045 |  | 0.103±0.051 |
| C3DC | 0.042±0.017 |  | 0.051±0.020 |  | 0.058±0.024 |
| C3DC/C10 | 0.446±0.321 |  | 0.416±0.316 |  | 0.450±0.278 |
| C18:1 | 0.455±0.130 |  | 0.924±0.294 |  | 0.982±0.353 |
| C18-OH | 0.016±0.008 |  | 0.014±0.006 |  | 0.017±0.006 |
| C10:1 | 0.078±0.047 |  | 0.103±0.060 |  | 0.119±0.076 |
| C5DC/C8 | 1.086±0.718 |  | 1.071±0.557 |  | 1.149±0.648 |
| (C0+C2+C3+C16+C18:1)/Cit | 2.045±0.648 |  | 2.564±0.861 |  | 3.017±1.051 |
| (C16+C18)/C0 | 0.048±0.016 |  | 0.044±0.012 |  | 0.038±0.011 |
| C0/(C16+C18) | 23.673±11.231 |  | 24.149±6.069 |  | 28.698±8.494 |
| (Glu+Gly)/(Cit+Arg) | 11.037±3.557 |  | 14.619±4.442 |  | 17.130±8.608 |
| Gln/Glu | 0.069±0.035 |  | 0.057±0.036 |  | 0.043±0.037 |
| Cit/Phe | 0.652±0.196 |  | 0.430±0.119 |  | 0.412±0.169 |
| Leu/Phe | 3.106±0.789 |  | 2.446±0.407 |  | 2.322±0.454 |
| C4-OH/C3DC | 1.181±0.708 |  | 1.208±0.825 |  | 1.205±0.574 |
| C3DC+C4-OH | 0.086±0.030 |  | 0.104±0.035 |  | 0.121±0.036 |
| (C16+C18:1)/C2 | 0.118±0.040 |  | 0.117±0.047 |  | 0.086±0.025 |
| Pro/Glu | 3.600±1.666 |  | 2.418±0.892 |  | 2.257±0.971 |

Abbreviation: AHU:asymptomatic hyperuricemia; Ala, Alanine; Arg, Arginine; Asn, Asparagine; Cit, Citrulline; Cys, Cysteine; Gln, Glutamine; Glu, Glutamic; Gly, Glycine; Hcy, Homocysteine; His, Histidine; Leu, Leucine; Lys, Lysine; Orn, Ornithine; Phe, Phenylalanine; Pip, Piperamide; Pro, Proline; Ser, Serine; Thr, Threonine; Trp, Tryptophan; Tyr, Tyrosine; Val, Valine; C0, Free carnitine; C2, Acetylcarnitine; C3, Propionylcarnitine; C4, Butyrylcarnitine; C4-OH, Hydroxybutyrylcarnitine; C4DC, Succinyl-/methylmalonylcarnitine; C5, Isovalerylcarnitine; C5-OH, 3-Hydroxyisovalerylcarnitine; C8, Octanoylcarnitine; C10, Decanoylcarnitine; C12, Lauroylcarnitine; C14, Myristoylcarnitine; C14-OH, 3-Hydroxyl-tetradecanoylcarnitine; C14DC, Tetradecanoyldiacylcarnitine; C14:1, Tetradecenoylcarnitine; C16, Palmitoylcarnitine; C16-OH, 3-Hydroxypalmitoylcarnitine; C16:1-OH, 3-Hydroxypalmitoleylcarnitine; C18, Octadecanoylcarnitine; C20, Arachidic carnitine; C22, Behenic carnitine; C24, Tetracosanoic carnitine.
